# Predictive model for the probability of malignancy in solitary pulmonary nodules: a meta-analysis

**DOI:** 10.1186/s13019-022-01859-x

**Published:** 2022-05-03

**Authors:** Gang Chen, Tian Bai, Li-Juan Wen, Yu Li

**Affiliations:** 1 Department of Radiology, Xuzhou Central Hospital, Xuzhou, China; 2 Radiological Imaging Diagnostic Center, The Third Affiliated Hospital of Qiqihar Medical University, Qiqihar, China

**Keywords:** Predictive model, Diagnosis, Solitary pulmonary nodule, Meta-analysis

## Abstract

**Background:**

To date, multiple predictive models have been developed with the goal of reliably differentiating between solitary pulmonary nodules (SPNs) that are malignant and those that are benign. The present meta-analysis was conducted to assess the diagnostic utility of these predictive models in the context of SPN differential diagnosis.

**Methods:**

The PubMed, Embase, Cochrane Library, CNKI, Wanfang, and VIP databases were searched for relevant studies published through August 31, 2021. Pooled data analyses were conducted using Stata v12.0.

**Results:**

In total, 20 retrospective studies that included 5171 SPNs (malignant/benign: 3662/1509) were incorporated into this meta-analysis. Respective pooled sensitivity, specificity, positive likelihood ratio (PLR), negative likelihood ratio (NLR), and diagnostic score values were 88% (95CI%: 0.84–0.91), 78% (95CI%: 0.74–0.80), 3.91 (95CI%: 3.42–4.46), 0.16 (95CI%: 0.12–0.21), and 3.21 (95CI%: 2.87–3.55), with an area under the summary receiver operating characteristic curve value of 86% (95CI%: 0.83–0.89). Significant heterogeneity among studies was detected with respect to sensitivity (I^2^ = 89.07%), NLR (I^2^ = 87.29%), and diagnostic score (I^2^ = 72.28%). In a meta-regression analysis, sensitivity was found to be impacted by the standard reference in a given study (surgery and biopsy vs. surgery only, P = 0.02), while specificity was impacted by whether studies were blinded (yes vs. unclear, P = 0.01). Sensitivity values were higher when surgery and biopsy samples were used as a standard reference, while unclear blinding status was associated with increased specificity. No significant evidence of publication bias was detected for the present meta-analysis (P = 0.539).

**Conclusions:**

The results of this meta-analysis demonstrate that predictive models can offer significant diagnostic utility when establishing whether SPNs are malignant or benign.

## Introduction

As thoracic computed tomography (CT) scans are routinely conducted during physical examinations for certain patient populations, solitary pulmonary nodules (SPNs) of uncertain clinical significance are relatively common clinical entities. These SPNs can be malignant or correspond to early-stage lung cancer [1], with an estimated 55–77% being malignant and with rising odds of malignancy with increasing SPN diameter [2–4].

While pathological diagnosis is generally the definitive approach to SPN differentiation, it necessitates invasive biopsy or surgical resection procedures. In order to avoid unnecessary invasive diagnostic procedures when possible, comprehensive alternative approaches to SPN evaluation are required [5–7]. Differential SPN diagnosis cannot be achieved successfully through the assessment of a single radiological or clinical feature, underscoring the need for the development of predictive models capable of gauging the likelihood that a given SPN is malignant. The first such predictive model was reported in 1999 by the Mayo clinic [8], with many more such models having been developed to date by multiple international research teams [9–30].

While promising, these predictive models exhibit significant variability among studies with respect to reported sensitivity and specificity values. These differences may be attributable to sample sizes and to whether models incorporated tumor marker tests or positron emission tomography (PET)/CT imaging results. There is thus a clear need for larger-scale analyses aimed at assessing the overall diagnostic value of these models.

As such, we herein conducted a meta-analysis designed to assess the diagnostic utility of predictive models used for the differential diagnosis of SPNs.

## Materials and methods

### Study selection

The PubMed, Embase, Cochrane Library, CNKI, Wanfang, and VIP databases were searched for relevant studies published as of August 31, 2021 using the following search strategy: ((((((diagnosis[Title/Abstract]) OR (analysis[Title/Abstract])) OR (probability[Title/Abstract])) OR (differential[Title/Abstract])) OR (predictive[Title/Abstract])) AND (model[Title/Abstract])) AND (((pulmonary nodule[Title/Abstract]) OR (lung nodule[Title/Abstract])) OR (SPN[Title/Abstract])). This meta-analysis was registered at https://inplasy.com/ (No. INPLASY2021100006).

Studies eligible for inclusion were: (1) studies assessing the differential diagnosis of benign and malignant SPNs; (2) studies of SPNs ≤ 30 mm in size; (3) studies in which predictive models were developed and provided; (4) studies in which sensitivity and specificity were provided. Studies were excluded if they were reviews, case reports, or non-human studies.

### Data extraction and quality assessment

Relevant data were independently extracted from included studies by two researchers, with any disagreements being resolved by a third researcher. Extracted data included authors, publication year, publication country, study design, blinding status, sample size, SPN size, reference standards, predictive model contents, and true-positive (TP), false-positive (FP), true-negative (TN), and false-negative (FN) results. Risk of bias was evaluated with the quality assessment of diagnostic accuracy studies (QUADAS-2) tool [31].

### Definitions

SPNs were defined as isolated round lung lesions ≤ 3 cm in size not associated with atelectasis, pleural effusion, or mediastinal lymphadenopathy [32]. A TP result was defined as one in which both the predictive model and final diagnosis were indicative of malignancy, while an FP result was defined as one in which the predictive model indicated that a lesion was malignant whereas the final diagnosis indicated that it was benign. A TN result was defined as one in which both the predictive model and final diagnosis were indicative of a benign SPN, while an FN result was defined as one in which a predictive model indicated that an SPN was benign but the final diagnosis for that lesion indicated it was malignant.

### Meta-analysis

The sensitivity, specificity, positive likelihood ratio (PLR), negative likelihood ratio (NLR), diagnostic score, and summary receiver operating characteristic (SROC) curve were pooled using Stata v12.0 (Stata Corporation, TX, USA).

A PLR > 5 or an NLR < 0.2 were considered to be indicative of high diagnostic ability for a given predictive model. Diagnostic ability was also considered to be good if the area under the SROC curve (AUC) was > 80% [33].

Heterogeneity was assessed with the I^2^ index, with an I^2^ > 50% being indicative of significant heterogeneity. A meta-regression was used to detect potential sources of heterogeneity, and subgroup analyses were conducted based upon these identified sources. Deeks’ funnel plots were used to gauge potential publication bias, and P < 0.05 was the threshold of significance for this study.

## Results

### Study selection

**Fig. 1:**
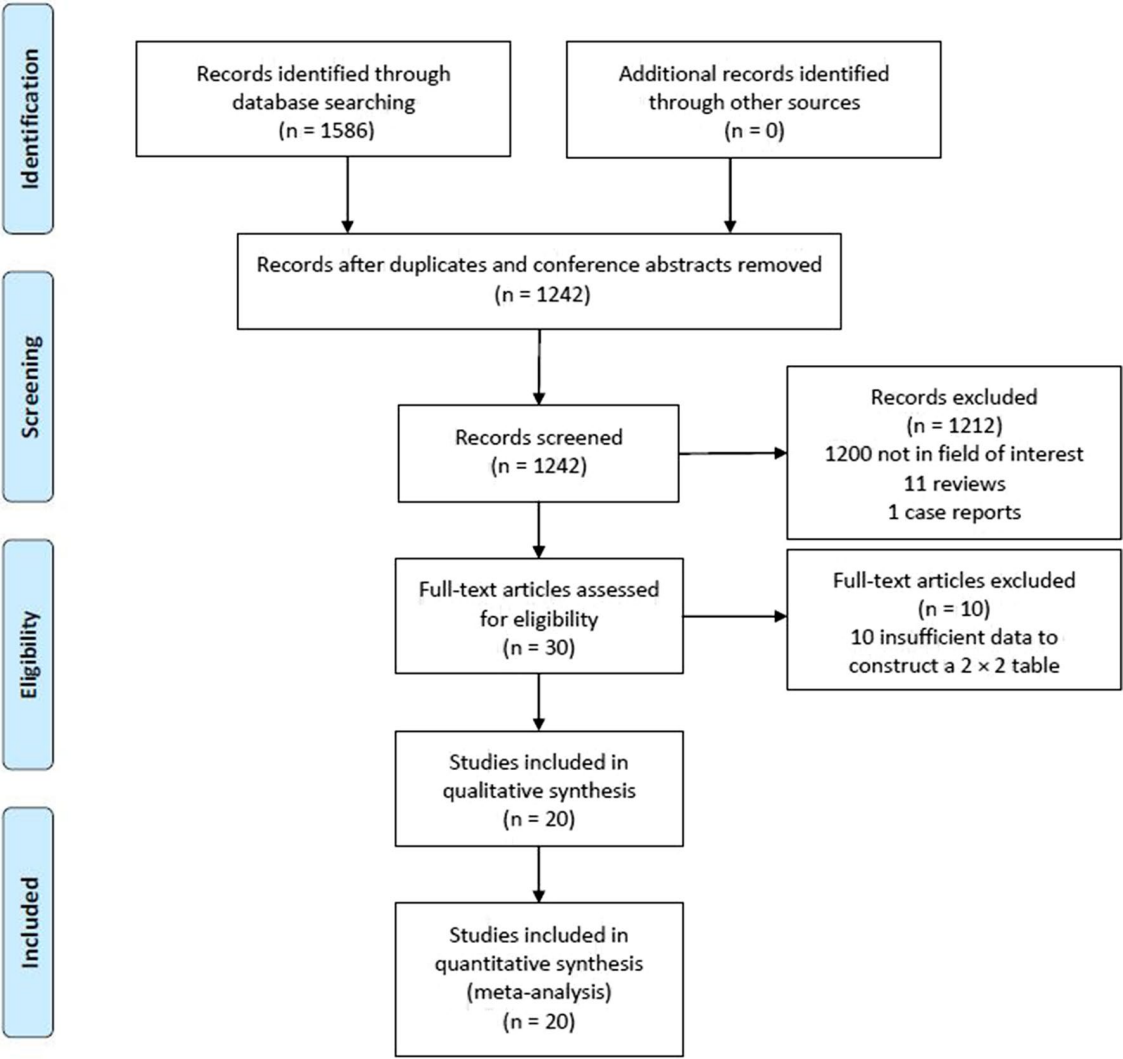
Flowchart diagram of our meta-analysis

The study selection process for the present study is outlined in Fig. 1. Ultimately, 20 studies were included in the final analysis, all of which were retrospective in design and conducted by Chinese research teams. We have found 4 eligible studies which were from out of China [7–10]. However, these studies had insufficient data to construct a 2 × 2 table, and therefore, these studies were excluded from this meta-analysis. These 20 studies (Table 1) included 5171 total SPNs (malignant: 3662; benign: 1509). PET/CT results were included in 7 of these studies [14, 15, 18–20, 22, 24], while 6 included tumor marker results [11, 16, 23, 24, 27, 29]. Moreover, 10 studies had predictive models consisting of > 4 factors [14–16, 20, 21, 23–27, 30]. The details of each predictive model are outlined in Table 2, and raw TP, FP, TN, and FN data are compiled in Table 3.

**Table 1:** Characteristics of studies included in meta-analysis

Studies	Year	Design	Blind	Sample size	M/B	Reference standard	PET/CT	Tumor markers
Cao [11]	2021	Retrospective	Unclear	80	55/25	S	No	Yes
Chen [12]	2020	Retrospective	Unclear	216	160/56	S	No	No
Chen [13]	2016	Retrospective	Unclear	289	207/82	S	No	No
Chen [14]	2013	Retrospective	Unclear	109	67/42	S, B	Yes	No
Cheng [15]	2019	Retrospective	Unclear	362	291/71	S, B	Yes	No
Dong [16]	2014	Retrospective	Unclear	1679	1296/383	S, B	No	Yes
Hu [17]	2016	Retrospective	Yes	112	82/30	S	No	No
Lin [18]	2015	Retrospective	Yes	186	123/63	S, B	Yes	No
Ma [19]	2020	Retrospective	Unclear	161	131/30	S, B	Yes	No
Tian [20]	2012	Retrospective	Unclear	105	61/44	S, B	Yes	No
Wang [21]	2018	Retrospective	Yes	268	156/112	S	No	No
Xiang [22]	2016	Retrospective	Yes	110	80/30	S	Yes	Yes
Xiao [23]	2019	Retrospective	Unclear	242	209/33	S, B	No	Yes
Xu [24]	2020	Retrospective	Unclear	160	122/38	S	Yes	Yes
Yang [25]	2012	Retrospective	Unclear	145	98/47	S	No	No
Yu [26]	2016	Retrospective	Unclear	139	73/66	S	No	No
Zhang [27]	2015	Retrospective	Unclear	120	72/48	S, B	No	Yes
Zhang [28]	2016	Retrospective	Unclear	270	110/160	S	No	No
Zhao [29]	2021	Retrospective	Yes	250	156/94	S, B	No	Yes
Zhong [30]	2017	Retrospective	Unclear	168	113/55	S, B	No	No

M: malignant; B: benign; S: surgery; B: biopsy; PET/CT: positron emission tomography/computed tomography

**Table 2:** The details of each predictive model

	Number of predictive factors	Items of predictive factors
Cao [11]	4	CEA, Cyfra211, previous tumor history, lobulation
Chen [12]	4	Density, concentrated vessel, nodule type, incisure
Chen [13]	7	Age, density, lesion-lung border, lobulation, concentrated vessel, pleural retraction, PET/CT
Chen [14]	7	Age, gender, calcification, lobulation, short spiculation, long spiculation, border
Cheng [15]	6	Age, vacuole, lobulation, calcification, diameter, PET/CT
Dong [16]	10	Age, CEA, Cyfra211, smoking, family tumor history, diameter, clear border, satellite lesions, lobulation, calcification, spiculation
Hu [17]	3	Solid component, diameter, concentrated vessel
Lin [18]	5	Age, lobulation, concentrated vessel, pleural retraction, PET/CT
Ma [19]	4	Age, concentrated vessel, calcification, PET/CT
Tian [20]	6	Age, smoking, gender, diameter, PET/CT, spiculation
Wang [21]	6	Gender, age, previous tumor history, GGN, diameter, spiculation
Xiang [22]	5	Age, PET/CT, lobulation, calcification, spiculation
Xiao [23]	6	Age, CEA, Cyfra211, consolidation tumor ratio > 50%, lobulation, calcification
Xu [24]	11	Age, tumor marker, family tumor history, diameter, border, lobulation, calcification, speculation, concentrated vessel, pleural retraction, GGN
Yang [25]	6	Age, family tumor history, diameter, clear border, spiculation, calcification
Yu [26]	8	Age, family tumor history, previous tumor history, clear border, lobulation, spiculation, air bronchogram, calcification
Zhang [27]	6	Age, Cyfra211, smoking, diameter, clear border, spiculation
Zhang [28]	3	Age, imaging feature, diameter
Zhao [29]	4	Age, CEA, pleural retraction, CT bronchus sign
Zhong [30]	8	Age, family tumor history, previous tumor history, clear border, lobulation, spiculation, pleural retraction, diameter

CEA: carcinoembryonic antigen; GGN: ground glass nodule; PET/CT: positron emission tomography/computed tomography

**Table 3:** Raw Data of diagnostic performance of studies included in this meta-analysis

	True positive	False positive	False negative	True negative
Cao [11]	39	2	16	23
Chen [12]	101	14	59	42
Chen [13]	168	17	49	67
Chen [14]	64	13	4	29
Cheng [15]	259	12	32	56
Dong [16]	1175	72	121	311
Hu [17]	77	12	5	18
Lin [18]	108	12	15	51
Ma [19]	129	7	2	23
Tian [20]	55	7	6	37
Wang [21]	125	34	31	78
Xiang [22]	69	6	11	24
Xiao [23]	183	10	26	23
Xu [24]	99	10	23	28
Yang [25]	93	16	5	31
Yu [26]	66	14	7	52
Zhang [27]	62	7	10	41
Zhang [28]	90	24	20	136
Zhao [29]	130	27	26	67
Zhong [30]	107	13	6	42

### Bias assessment

**Fig. 2:**
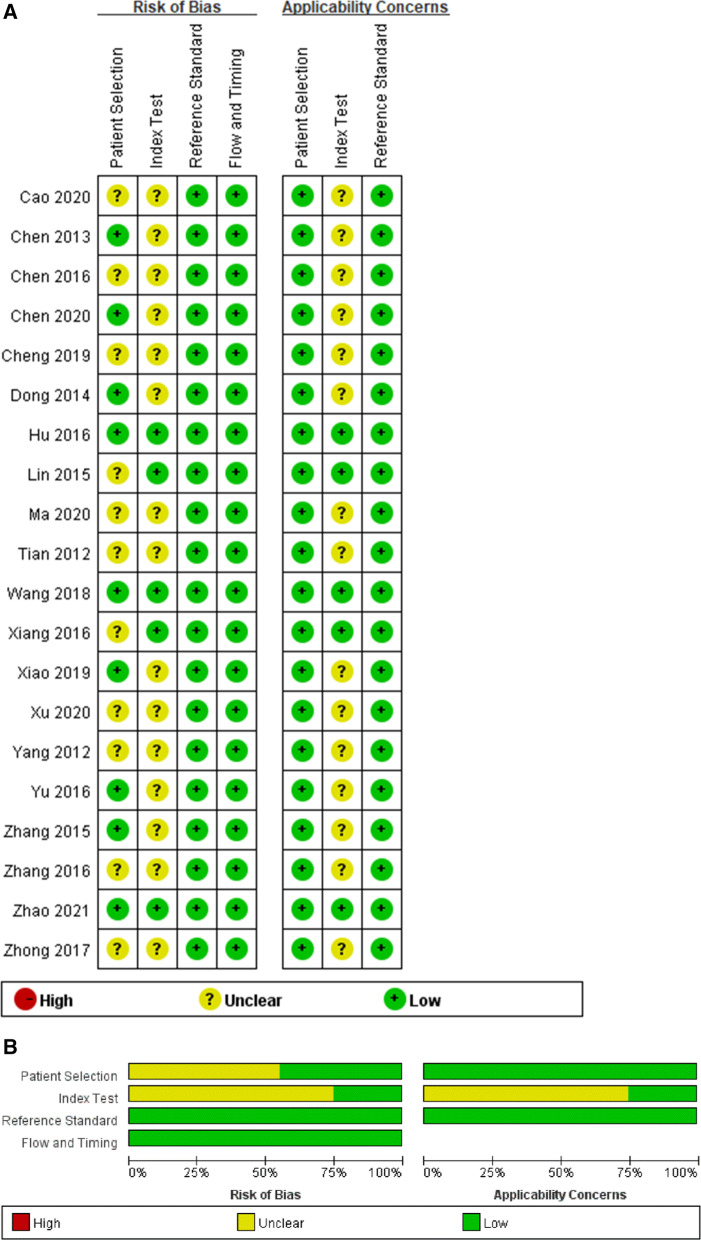
Representation of the methodological quality **A** Graph and **B** Summary

The QUADAS-2 tool was used to assess potential bias in the present meta-analysis (Fig. 2). Of the 20 included studies, 11 failed to indicate whether patients were enrolled in a consecutive manner [11, 13, 15, 18–20, 22, 24. 25, 28, 30], while 14 provided unclear information pertaining to the blinding status [11–16, 19, 20, 23–28, 30]. All studies described the reference standard used to confirm the diagnosis.

### Diagnostic results

**Fig. 3:**
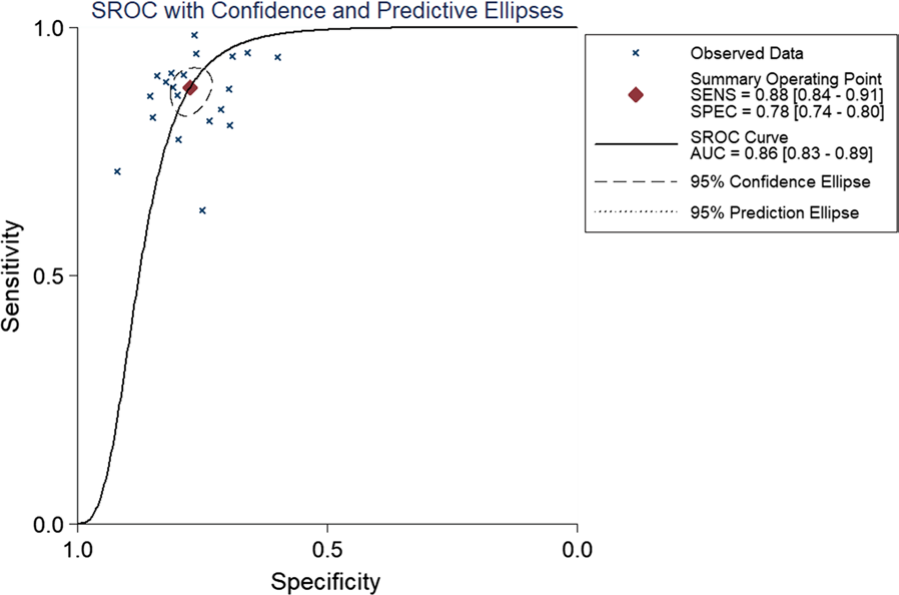
SROC in this meta-analysis

Pooled sensitivity, specificity, PLR, NLR, and diagnostic score values were 88% (95CI%: 0.84–0.91, Table 4), 78% (95CI%: 0.74–0.80, Table 4), 3.91 (95CI%: 3.42–4.46, Table 4), 0.16 (95CI%: 0.12–0.21, Table 4), and 3.21 (95CI%: 2.87–3.55, Table 4), respectively. Significant heterogeneity was detected with respect to sensitivity (I^2^ = 89.07%), NLR (I^2^ = 87.29%), and diagnostic score (I^2^ = 72.28%). The AUC value was 86% (95CI%: 0.83–0.89, Fig. 3). The SROC curve consistent with substantial deviation from a shoulder-like appearance, exhibiting the potential absence of any threshold effect.

**Table 4:** Results of this meta-analysis and the subgroup analyses

	Studies (n)	Sensitivity (95% CI)	Specificity (95% CI)	PLR (95% CI)	NLR (95% CI)
All studies	20	88% (84–91%)	78% (74–80%)	3.91 (3.42–4.46)	0.16 (0.12–0.21)
Reference standard					
Surgery only	10	84% (77–89%)	77% (71–81%)	3.56 (2.92–4.36)	0.22 (0.15–0.30)
Surgery and biopsy	10	91% (87–93%)	79% (75–82%)	4.29 (3.59–5.14)	0.12 (0.09–0.16)
Blind					
Yes	5	86% (81–90%)	73% (67–78%)	3.14 (2.56–3.85)	0.19 (0.14–0.26)
Unclear	15	88% (83–92%)	80% (76–83%)	4.33 (3.73–5.02)	0.15 (0.10–0.21)

PLR: positive likelihood ratio; NLR: negative likelihood ratio; CI: confidence interval

The results of a meta-regression analysis are shown in Table 5. Sensitivity was impacted by the reference standards utilized in a given study (surgery only vs. surgery and biopsy, P = 0.02). Specificity was impacted by whether blinding was employed (yes vs. unclear, P = 0.01). Sample size, the number of predictive factors, whether models incorporated PET/CT results, and whether models incorporated tumor marker results had no impact on the final diagnostic results.

**Table 5:** Results of meta-regression

	Sensitivity			Specificity		
	Estimate	Coefficient	P value	Estimate	Coefficient	P value
Publication year	0.91 (0.85–0.95)	2.35	0.12	0.79 (0.73–0.84)	1.32	0.51
Sample size	0.89 (0.85–0.92)	2.11	0.24	0.77 (0.72–0.80)	1.19	0.45
Reference standard	0.91 (0.87–0.94)	2.30	0.02	0.78 (0.74–0.82)	1.28	0.58
Number of predictive factors	0.86 (0.79–0.91)	1.80	0.40	0.78 (0.73–0.83)	1.27	0.76
Blind	0.88 (0.84–0.92)	2.01	0.77	0.80 (0.76–0.83)	1.36	0.01
Whether contained PET/CT	0.86 (0.81–0.90)	1.82	0.17	0.77 (0.73–0.81)	1.22	0.66
Whether contained tumor markers	0.89 (0.85–0.92)	2.10	0.25	0.77 (0.73–0.80)	1.20	0.49

PET/CT: positron emission tomography/computed tomography

Subgroup analyses were conducted based upon differences in reference standard and blinding situations (Table 4). Higher sensitivity was observed in the subgroup in which surgery and biopsy were used for reference sample collection, while higher specificity was evident in the subgroup in which the blinding situation was unclear.

### Publication bias

Deeks’ funnel plot asymmetry test did not reveal any evidence of publication bias in the present meta-analysis (P = 0.539).

### Lobulation sign

We found that lobulation sign was the most common feature of the predictive models and it occurred in 11 of the 20 studies [11, 13–16, 18, 22–24, 26, 30]. The TP, FP, TN, and FN data of lobulation sign could be extracted from 7 studies [11, 16, 22–24, 26, 30]. Pooled sensitivity, specificity, PLR, NLR, and diagnostic score values were 57% (95CI%: 0.38–0.74), 80% (95CI%: 0.62–0.91), 2.84 (95CI%: 1.76–4.63), 0.54 (95CI%: 0.40–0.72), and 1.66 (95CI%: 1.17–2.16), respectively. The AUC value was 74% (95CI%: 0.70–0.78).

## Discussion

In this meta-analysis, we explored the diagnostic utility of predictive models in the context of SPN differential diagnosis. Overall, we found these models to exhibit robust predictive value with a high AUC value of 86%. As the NLR value (0.16) was less than 0.2, this indicated that lower predictive scores were associated with the satisfactory prediction of benign SPNs. However, the pooled PLR value (3.91) was less than 5, indicating that higher predictive scores were only moderately predictive of malignant SPNs.

In CT-based analyses, spiculation and calcification signs are commonly leveraged as predictive factors when evaluating SPNs, and are routinely incorporated into developed predictive models [32, 33]. Spiculation sign is generally indicative of malignant SPNs, whereas calcified nodules are more likely to be benign. However, one prior meta-analysis found spiculation sign to only exhibit moderate diagnostic accuracy when used to evaluate SPNs (AUC = 76%) [33]. In a separate meta-analysis, calcification was found to be a good predictor of benign SPN status (PLR = 6.06) [32], although the overall diagnostic utility of such calcification was somewhat limited (AUC = 65%) [32]. These results suggest that any individual sign only offers limited diagnostic value in the evaluation of SPNs. Combining these signs together, however, may significantly improve the overall diagnostic value of developed models.

Sensitivity values were impacted by the reference standard used in included studies (P = 0.02), with higher sensitivity values being reported when the references standard included surgery and biopsy samples. While benign are SPNs are generally confirmed via surgical resection, malignancy SPNs can be confirmed via both surgery and biopsy [34]. When researchers only focused on surgically-confirmed SPNs in their studies, this markedly reduced the malignant SPN sample size, thereby constraining the sensitivity of the resultant models.

Specificity values were found to be impacted by the blinding situation for included studies (P = 0.01), with an unclear blinding situation being associated with higher specificity. This may be attributable to the relatively high number of studies with unclear blinding (n = 15) as compared to the number of studies with definitive blinding (n = 5).

In one prior meta-analysis, tests for carcinoembryonic antigen (CEA) alone were found to be associated with moderate diagnostic utility (AUC = 77%) when differentiating between malignant and benign SPNs [35]. However, the incorporation of other tumor marker tests can improve the overall accuracy of developed diagnostic models for SPN evaluation [24]. PET/CT also exhibits high diagnostic ability when used to assess SPNs [36]. Even so, in the present meta-analysis, the incorporation of tumor marker and PET/CT tests did not increase the overall diagnostic utility of developed predictive models. This may be attributable to the fact that there were relatively few studies that included tumor marker (n = 6) and PET/CT (n = 7) tests in the overall analysis.

In this meta-analysis, lobulation sign was the most common feature of the included predictive models. However, the area under the SROC curve was only 74%, which was less than that (86%) made by the predictive models. This finding indicated that predictive model could provide more comprehensive analysis for SPNs than a single feature did.

There are certain limitations to this meta-analysis. For one, the major limitation is the fact that all included studies were retrospective nature, and this caused the major bias in the results of this meta-analysis. Additional prospective studies will thus be critical to validate and expand these results. Secondly, many of these studies failed to clarify whether consecutive patients were enrolled, potentially influencing the diagnostic accuracy of the developed predictive models. Third, none of these studies employed a CT-based follow-up approach to confirm the identity of SPNs that were diagnosed as benign. While surgical resection can provide the most precise diagnostic information pertaining to benign lesions, CT follow-up can also be accepted for final diagnosis [37]. The absence of CT-based follow-up may have thus impacted the reported diagnostic accuracy. Fourth, none of these models included magnetic resonance imaging (MRI)-based results. While MRI scans are not commonly used to evaluate lung disease, some prior studies have suggested that they may offer value as a means of distinguishing between SPNs that are malignant and those that are benign [38]. Lastly, all included studies were from China and this may further increase the risk of bias. Although China is the country with the world largest population, additional studies from other countries are still needed.

## Conclusion

In summary, this meta-analysis demonstrated that predictive models offer substantial diagnostic value when establishing whether SPNs are malignant or benign, although further research will be required to confirm these findings.

## Data Availability

The data that support the findings of this study are available from the corresponding author upon reasonable request.

## Abbreviations

CEA: Carcinoembryonic antigen
CT: Computed tomography
FN: False negative
FP: True negative
PLR: Positive likelihood ratio
NLR: Negative likelihood ratio
SROC: Summary receiver operating characteristic
TN: True negative
TP: True positive
MRI: Magnetic resonance imaging
SPN: Solitary pulmonary nodules
